# Associação Causal entre Células Imunes Mediadas por Metabólitos Plasmáticos e Infarto do Miocárdio: Um Estudo de Randomização Mendeliana

**DOI:** 10.36660/abc.20250026

**Published:** 2026-04-28

**Authors:** Yue Liu, Xing Chen, Yingzhi Liu, Yvwen Lu, Yun Zhu, Lingli Chen, Jie Li

**Affiliations:** 1 Hunan University of Chinese Medicin Changsha Hunan China Hunan University of Chinese Medicin, Changsha, Hunan – China

**Keywords:** Análise da Randomização Mendeliana, Biomarcadores, Infarto do Miocárdio

## Abstract

**Fundamento:**

O infarto do miocárdio (IM), um evento cardiovascular agudo, tem sido associado, em estudos anteriores, a respostas imunes e metabólitos. No entanto, a relação causal entre células imunes e IM, bem como o papel mediador dos metabólitos plasmáticos, ainda não foram claramente confirmados.

**Objetivos:**

Análise sistemática da associação causal entre 731 células imunes e IM, bem como os papéis mediadores de 1400 metabólitos plasmáticos.

**Métodos:**

A análise de randomização mendeliana (RM) bidirecional foi utilizada para explorar a relação causal entre células imunes e IM, e análises de mediação em uma e duas etapas foram realizadas para identificar metabólitos plasmáticos. Os métodos de análise incluíram variância inversa ponderada (VIP) (p<0,05), mediana ponderada (p<0,05), moda simples (p<0,05), moda ponderada (p<0,05), regressão MR-Egger (p<0,05), intercepto MR-Egger (p>0,05) e MR-PRESSO (p>0,05) para avaliar a confiabilidade dos resultados. Os SNPs associados a células imunes e metabólitos foram submetidos à seleção de genes utilizando o banco de dados STRING, seguida por análise de enriquecimento.

**Resultados:**

Um total de 44 células imunes foram identificadas com associação causal com o IM (p<0,05), dentre as quais o HLA DR em células dendríticas (ebi-a-GCST90002106) apresentou a significância mais forte (p<0,001). Um total de 19 SNPs foram identificados (OR=1,038, IC 95% 1,018-1,059). A análise reversa não mostrou significância estatística (p>0,05). Metabólitos plasmáticos, incluindo os níveis de glicuronídeo de ácido deoxicólico, a razão fosfato/N-acetilneuraminato e a glicosilceramida, desempenharam um papel mediador entre as células imunes e o IM, com efeitos de mediação de 7,0%, 4,6% e 6,6%, respectivamente. A análise do banco de dados STRING identificou oito genes-chave e uma via de sinalização que potencialmente conectam os fenótipos imunes e os três metabólitos (p<0,05).

**Conclusões:**

Este estudo identificou uma relação causal entre células imunes e IM. Os metabólitos níveis de glicuronídeo de ácido deoxicólico, razão fosfato/N-acetilneuraminato e glicosilceramida desempenharam um papel mediador parcial nessa relação causal.

## Graphical abstract

**Figura Central:**
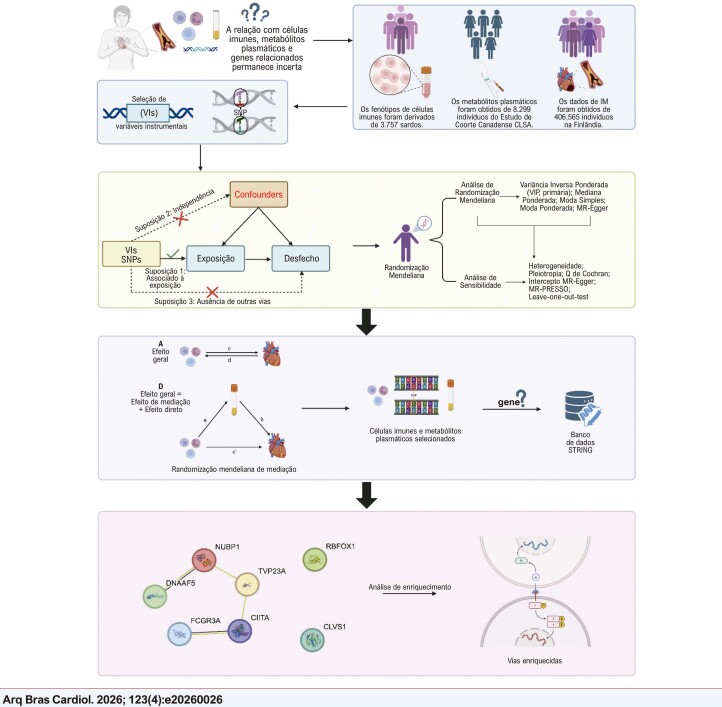
: Associação Causal entre Células Imunes Mediadas por Metabólitos Plasmáticos e Infarto do Miocárdio: Um Estudo de Randomização Mendeliana

*Relação causal entre células imunes e infarto do miocárdio e análise da mediação de metabólitos associados (Figura criada usando o BioRender).*

## Introdução

As doenças cardiovasculares são uma das principais causas de morte em todo o mundo, sendo a doença cardíaca isquêmica responsável por 49,2% desses óbitos. O infarto do miocárdio (IM) é tipicamente causado pela obstrução de artérias ou enxertos de bypass por trombose, caracterizado por uma redução súbita do fluxo sanguíneo para o miocárdio, levando, em última instância, à insuficiência cardíaca e à morte miocárdica. Apesar dos avanços significativos no tratamento do IM, ele permanece uma doença globalmente prevalente, com alta morbidade e mortalidade. Embora os métodos de tratamento tenham melhorado, muitos desafios persistem na identificação de uma estratégia terapêutica ideal.^1^ Durante o IM, ocorre morte celular miocárdica, o tecido na área infartada sofre necrose e uma resposta inflamatória é ativada. As células imunes podem tanto promover a morte celular miocárdica e a inflamação, quanto facilitar a regeneração do miocárdio danificado, tornando-se um componente importante na pesquisa da doença.^2^ A remodelação miocárdica após o IM é um processo complexo de reparo caracterizado pela infiltração de vários tipos de células imunes.^3^ Doenças metabólicas relacionadas, como hipercolesterolemia, hipertrigliceridemia, diabetes tipo 2 e hipertensão, estão associadas à ativação de células imunes. Esses fatores podem interferir no processo inflamatório de cicatrização após o IM. É evidente que existe uma interação complexa entre células imunes e metabólitos, e essa relação intrincada pode contribuir conjuntamente para a ocorrência e progressão do IM. Isso também sugere que os metabólitos podem atuar como mediadores, influenciando as células imunes e o desenvolvimento da doença. Explorar o papel mediador dos metabólitos plasmáticos na relação entre células imunes e IM pode aprofundar a compreensão geral do IM e fornecer novos alvos terapêuticos e direções de pesquisa.

A análise de randomização mendeliana (RM) é uma técnica estatística que utiliza variações genéticas como variáveis instrumentais (VIs) para explorar relações causais entre fatores de exposição e desfechos. Esse método utiliza efetivamente os resultados de estudos de associação genômica ampla (GWAS), empregando variações genéticas como VIs para investigar a relação causal entre exposição e desfecho.^4^ Atualmente, há uma carência de pesquisas sobre a relação causal entre células imunes mediadas por metabólitos plasmáticos e IM. Este estudo visa utilizar o método de RM para explorar a relação causal entre células imunes e IM, analisar a correlação entre metabólitos relevantes e IM, e revelar como as células imunes influenciam o início e a progressão do IM por meio de vias metabólicas específicas no plasma. Espera-se que os resultados deste estudo forneçam suporte teórico para estratégias de tratamento clínico baseadas na regulação metabólica, promovam o desenvolvimento de novas abordagens terapêuticas e, em última análise, melhorem o prognóstico de pacientes com IM.

## Materiais e métodos

### Desenho do estudo

Este estudo adota uma análise de RM de mediação em duas etapas para avaliar o papel dos metabólitos plasmáticos na regulação do IM pelas células imunes. Primeiramente, por meio de uma análise de RM bidirecional de duas amostras, as células imunes são tratadas como fatores de exposição e o IM como variável de desfecho para avaliar a relação causal entre 731 células imunes e o IM, e para derivar o efeito total (beta_all). Simultaneamente, quando o IM é utilizado como fator de exposição e as 731 células imunes como variável de desfecho, uma análise de RM reversa é realizada para garantir que nenhuma relação causal seja encontrada. Em seguida, 47 células imunes relacionadas ao IM são selecionadas. Por meio de uma análise de RM de duas amostras, a relação causal entre as células imunes mais significativamente associadas e 1400 metabólitos plasmáticos é analisada para derivar o tamanho do efeito (beta1) dos metabólitos mediadores. Posteriormente, os metabólitos plasmáticos selecionados são tratados como fatores de exposição e o IM como variável de desfecho para avaliar ainda mais a relação causal entre eles e derivar o beta2. Combinando beta_all, beta1 e beta2, realizamos uma análise de mediação para avaliar o papel mediador dos metabólitos plasmáticos na regulação do IM pelas células imunes, avaliando sistematicamente seu impacto. Por fim, integramos e analisamos sistematicamente os SNPs das células imunes e dos metabólitos, utilizando o banco de dados STRING para identificar os genes relacionados. Posteriormente, realizamos uma análise de enriquecimento para selecionar as vias envolvidas e construímos uma rede de interação para identificar nós-chave e vias potenciais. Essa abordagem revela de forma abrangente os potenciais mecanismos de interação entre células imunes e metabólitos no desenvolvimento e progressão de doenças, fornecendo uma base teórica e direcionamento para estudos de intervenção subsequentes.

### Fontes de dados

Os dados para este estudo foram obtidos a partir das estatísticas resumidas do GWAS (Estudo de Associação Genômica Ampla) de 731 fenótipos de células imunes (GCST90001391-GCST90002121) disponíveis no Catálogo GWAS.^5^ Dentre as 731 células imunes, os dados foram obtidos por meio de análise de citometria de fluxo. Destes, 118 representam contagens celulares absolutas (CA), 389 refletem a intensidade de fluorescência mediana, 32 são parâmetros morfológicos e 192 representam contagens celulares relativas. Especificamente, essas células foram cuidadosamente classificadas em sete grupos principais, incluindo células B, células dendríticas (CDs) clássicas, células T maduras, monócitos, células derivadas de mieloides, linfócitos TBNK (células T, células B, células natural killer) e células T reguladoras (Treg). Os dados originais do GWAS com características imunes foram derivados de 3757 indivíduos não relacionados da população da Sardenha, sem sobreposição entre as coortes. Aproximadamente 22 milhões de genótipos de SNPs de alta densidade foram inseridos e, após o ajuste para fatores de confusão (como sexo, idade, etc.), a análise de correlação foi realizada com base no painel de referência da Sardenha.^6^ Os dados de GWAS sobre metabólitos circulantes utilizados neste estudo incluíram informações de 8.299 indivíduos e abrangeram um total de 1.400 metabólitos, compreendendo 1.091 metabólitos e 309 razões de metabólitos, dos quais 850 foram identificados como metabólitos conhecidos. Esses dados foram derivados da coorte do *Canadian Longitudinal Study on Aging* (CLSA), que representa um importante recurso para GWAS metabolômicos e fornece informações valiosas para investigar mecanismos metabólicos e suas associações com a variação genética. Todos os dados estão disponíveis publicamente, com números de acesso variando de GCST90199621 a GCST90201020. Os dados de IM para este estudo foram obtidos do banco de dados FinnGen, com o número de identificação de dados I9_MI_STRICT. Este banco de dados contém dados genômicos em larga escala da Finlândia, que foram submetidos a uma triagem rigorosa e controle de qualidade para garantir a representatividade das amostras e a precisão dos dados. O tamanho total da amostra é de 406.565 indivíduos, incluindo 378.019 controles e 28.546 casos da doença. A coleta de dados abrange indivíduos de diferentes idades, sexos e históricos clínicos. Como este estudo utiliza dados resumidos disponíveis publicamente e previamente publicados, nenhuma aprovação ética adicional é necessária. Todos os dados originais foram obtidos com o consentimento informado dos participantes. Todos os dados utilizados neste estudo foram obtidos de GWAS ou estatísticas resumidas do FinnGen disponíveis publicamente. Nenhuma amostra em nível individual foi acessada ou retida neste estudo. Portanto, o tamanho da amostra foi determinado por todos os dados disponíveis nos conjuntos de dados originais do GWAS ou do FinnGen.

### Seleção de variáveis instrumentais

Os SNPs são usados como uma ferramenta poderosa para avaliar associações potenciais. Esses dados de SNPs são tipicamente provenientes de GWAS e são considerados VIs.^7^ Neste estudo, os SNPs são usados como VIs para avaliar relações causais. O delineamento do estudo de RM precisa satisfazer três pressupostos básicos: (1) as VIs estão fortemente associadas à exposição; (2) as VIs são independentes de quaisquer fatores de confusão; (3) as VIs afetam o desfecho exclusivamente por meio da exposição.^8^ Considerando que o uso do limiar de significância tradicional de $P<5\times 10^{-8}$ pode limitar o número de SNPs disponíveis no estudo, afetando assim a viabilidade da análise, este estudo adotou um limiar de $P<1\times 10^{-5}$ para aumentar o número de SNPs disponíveis para análise. Essa abordagem aumenta efetivamente o número de SNPs utilizáveis, aumentando assim o poder estatístico da análise e garantindo a confiabilidade e abrangência dos resultados. Para remover o desequilíbrio de ligação (DL), foi aplicado um limiar de (kb=10.000, r^2^=0,001). A força dos instrumentos foi avaliada usando a fórmula da estatística F: $F=\left[R^{2}\times (N-1-K)\right]/\left[K\times \left(1-R^{2}\right)\right]$ , onde R^2^ representa a proporção da variância na exposição explicada pela variação genética, N representa o tamanho da amostra do GWAS de exposição e K representa o número de SNPs. Se a estatística F correspondente for >10, isso indica ausência de viés de instrumento fraco significativo, aumentando assim a confiabilidade dos resultados da análise de RM.^9^

### Métodos estatísticos

Todas as análises estatísticas e gráficos foram realizados utilizando os softwares R4.4.0 e RStudio, com o pacote “TwosampleMR” e outros utilizados para as análises de RM e mediação. Um valor de p < 0,05 foi considerado estatisticamente significativo. Este estudo empregou cinco métodos diferentes para avaliar os efeitos causais bidirecionais, incluindo variância inversa ponderada (VIP), mediana ponderada (MP), moda simples, moda ponderada e regressão MR-Egger. Como a VIP é considerada robusta na inferência causal, foi escolhida como o método principal para estimar os efeitos causais. Para abordar possíveis problemas, como pleiotropia, foram realizadas análises de regressão MR-Egger e validação MR-PRESSO. A heterogeneidade foi avaliada utilizando o teste Q de Cochran, a análise de sensibilidade foi conduzida utilizando a análise leave-one-out e os potenciais vieses foram avaliados por meio de gráficos de funil, particularmente para verificar pleiotropia e viés de publicação. Utilizamos a Razão de Chances (OR) e o respectivo intervalo de confiança (IC) de 95% para quantificar e descrever com precisão a magnitude do efeito e sua incerteza. A OR, como medida estatística, reflete claramente a probabilidade relativa de um evento ocorrer entre dois ou mais grupos, enquanto o IC de 95% oferece a margem de confiabilidade da estimativa da OR, aprimorando ainda mais a interpretabilidade e a credibilidade dos resultados do estudo.

### Análise de mediação

Um delineamento de RM em duas etapas foi utilizado para a análise de mediação, a fim de investigar se os metabólitos plasmáticos mediam a via causal entre as células imunes e o IM. Primeiramente, para avaliar a relação causal entre as células imunes e o IM, foi aplicada uma análise de RM com duas amostras. O objetivo desta etapa foi verificar a associação entre as células imunes e o IM e calcular o efeito geral (c). Em seguida, uma análise de RM reversa foi conduzida, com o IM como fator de exposição e as células imunes como variável de desfecho, para examinar mais detalhadamente se existe uma relação causal entre as células imunes e o IM.

**Figura 1:**
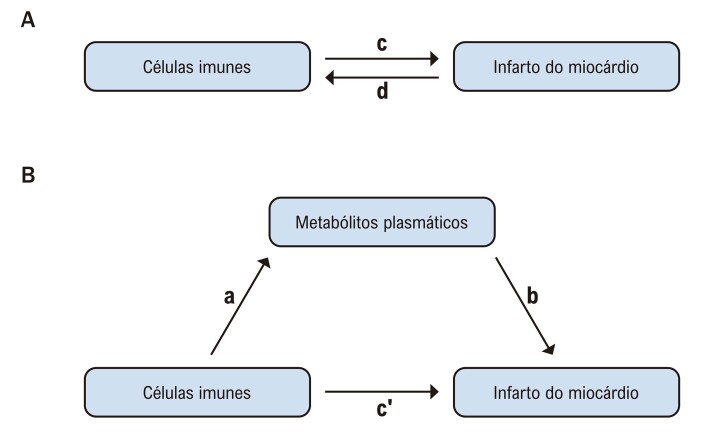
– (A) Efeito geral entre células imunes e infarto do miocárdio (IM): c representa o efeito geral usando células imunes como exposição e IM como resultado, enquanto d representa o efeito total usando IM como exposição e células imunes como resultado. (B) Decomposição do efeito geral em: (1) Efeito de mediação (ab) usando o método de duas etapas, onde a representa o efeito das células imunes sobre os metabólitos e b representa o efeito dos metabólitos sobre o IM. (2) Efeito direto ( $c^{\prime }=c-a\times b$ ).

Analisamos se os metabólitos plasmáticos mediam a relação entre as células imunes e os desfechos de IM, empregando um delineamento de RM em duas etapas para explorar ainda mais esse efeito mediador. A análise de RM de mediação inclui o efeito a, o efeito b e o efeito mediador (ab), com o efeito direto ( $c^{\prime }=c-a\times b$ ) calculado. O efeito direto se refere à influência das células imunes no IM, enquanto o efeito mediador reflete o impacto das células imunes no IM por meio da mediação dos metabólitos plasmáticos. Finalmente, comparando o efeito indireto e o efeito total, calculamos a porcentagem do efeito mediador ( Figura 1 ).^10 , 11^

### Resultados da análise de RM

#### Relação causal entre 731 células imunes e infarto do miocárdio

**Figura 2:**
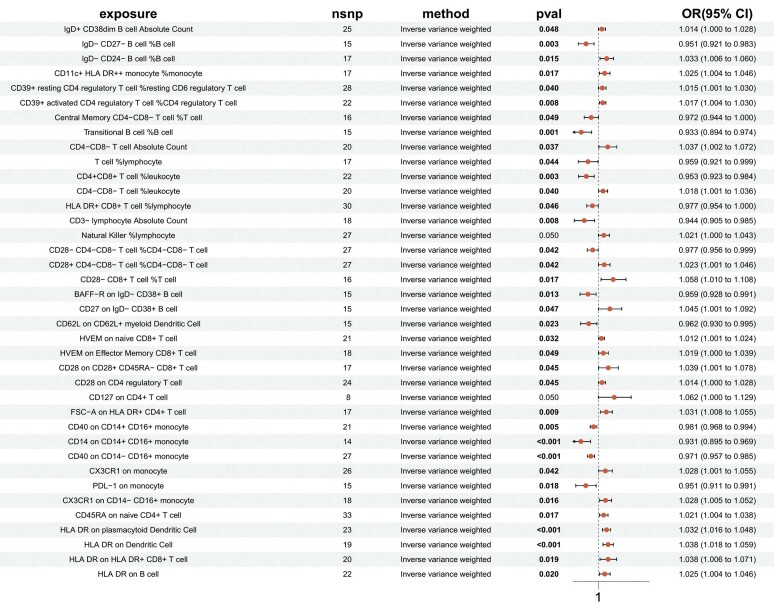
– Resultados de RM de células imunes e IM (Método VIP).

**Figura 3:**
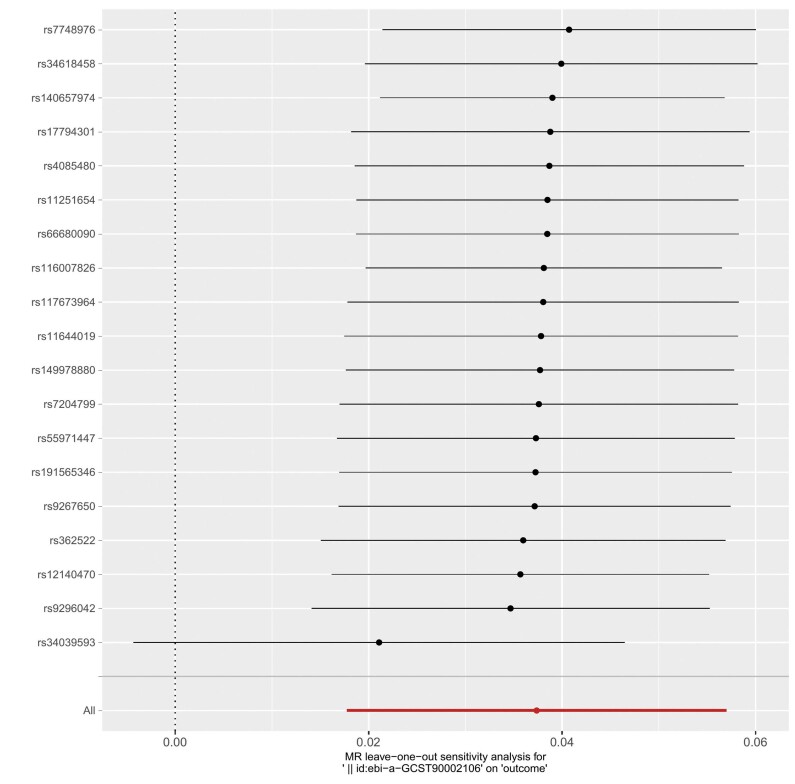
– Resultados da análise de sensibilidade de RM do HLA DR em células dendríticas e IM.

**Figura 4:**
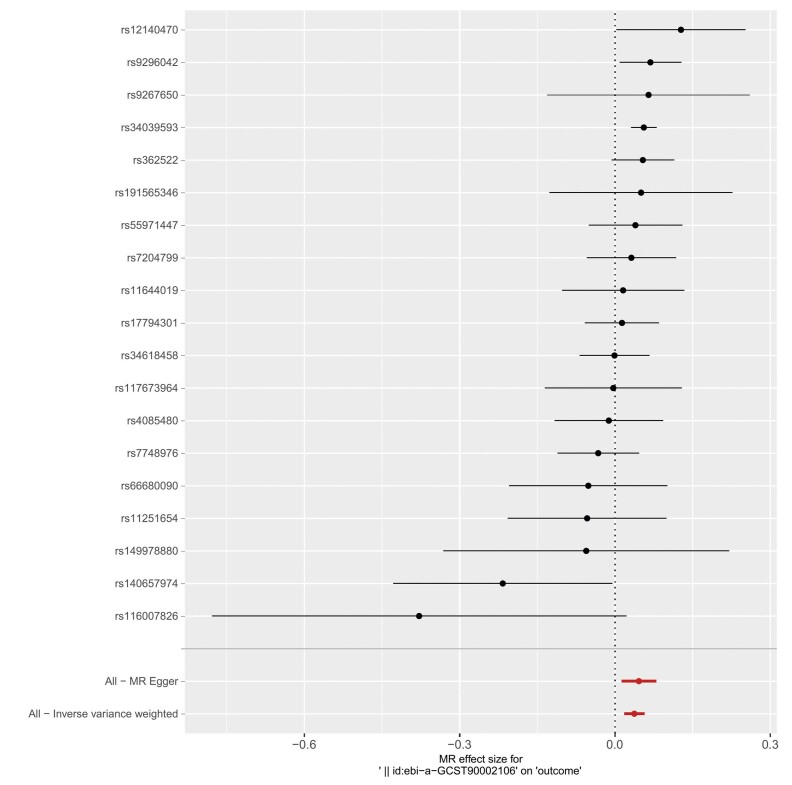
– Resultados do gráfico de floresta da análise RM entre HLA DR em células dendríticas e IM.

Neste estudo, para explorar a relação causal entre células imunes e IM, 731 fenótipos de células imunes foram utilizados como fatores de exposição e o IM como variável de desfecho. Foram realizadas 731 análises de RM de duas amostras, após a remoção do DL e de VIs fracas. Os resultados revelaram que 47 fenótipos de células imunes estavam causalmente associados ao IM. A análise de sensibilidade identificou 9 fenótipos de células imunes com heterogeneidade e pleiotropia horizontal, e esses 9 fenótipos foram removidos. Após a remoção, a análise VIP mostrou que 38 fenótipos de células imunes apresentavam relações causais confiáveis com o IM ( Figura 2 ). Dentre esses, o fenótipo de célula imune HLA DR em Célula Dendrítica (ebi-a-GCST90002106) foi o fator de risco mais significativo para IM. A relação causal entre HLA DR em CDs e IM não apresentou heterogeneidade ou pleiotropia horizontal, conforme indicado pelo teste Q de Cochran, teste MR-PRESSO e teste de intercepto MR-Egger. Os resultados da análise RM reversa de HLA DR em CDs e IM (método VIP, p>0,05) não mostraram significância estatística, indicando que não há relação causal reversa entre metabólitos plasmáticos e IM. A análise de sensibilidade RM, o gráfico de floresta e o gráfico de dispersão para HLA DR em CDs e IM são mostrados nas Figuras 3 , 4 e 5 .

**Figura 5:**
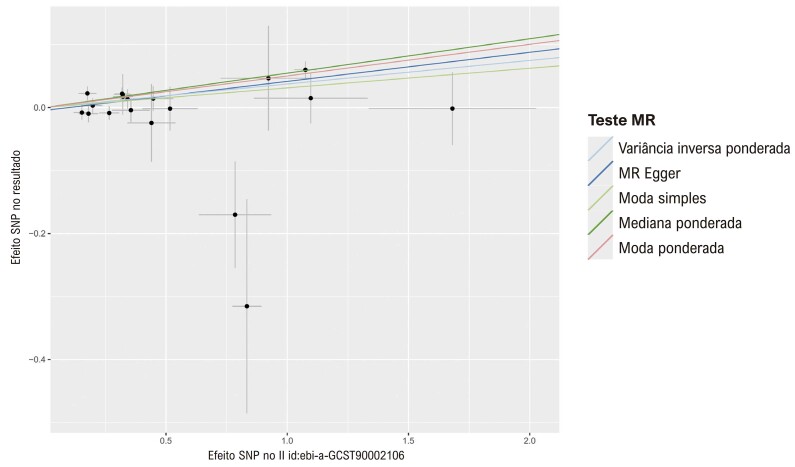
– Gráfico de dispersão dos resultados da análise de RM entre HLA DR em células dendríticas e IM.

**Figura 6:**
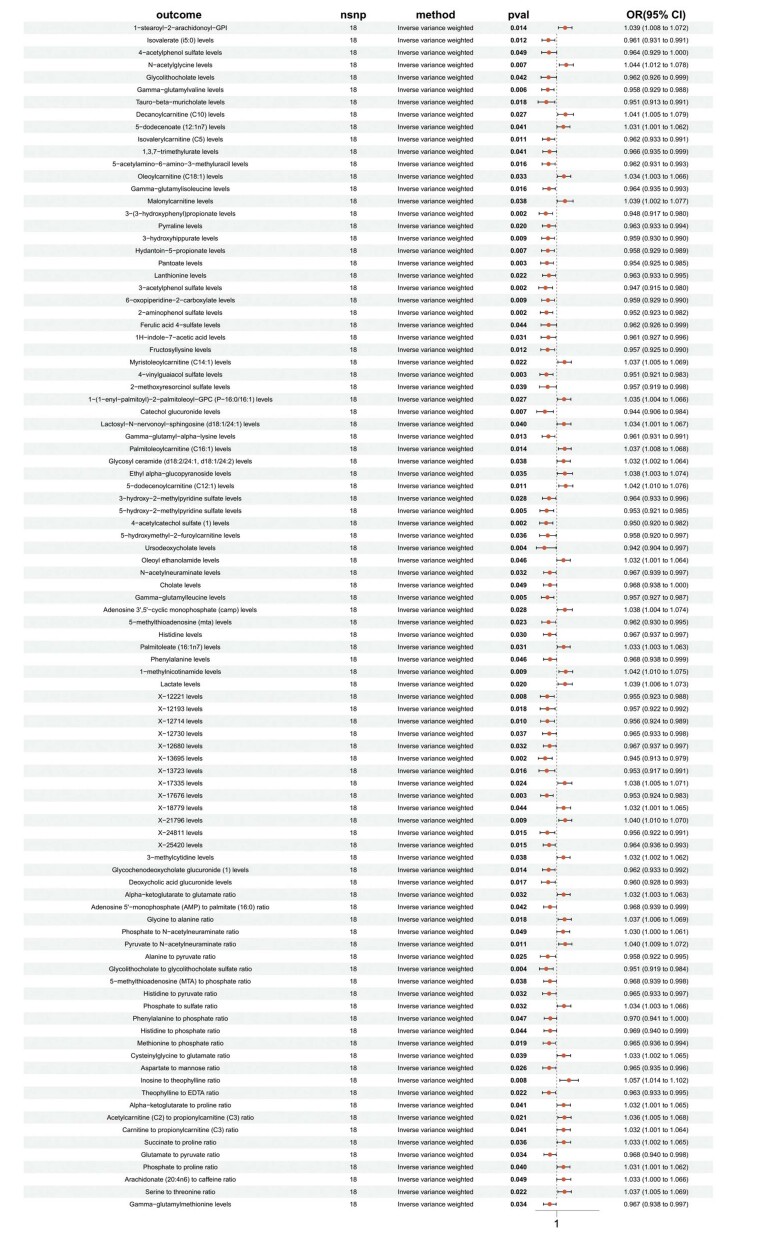
– Resultados da análise de HLA DR em células dendríticas com 1.400 metabólitos plasmáticos.

## Análise de RM de 96 metabólitos plasmáticos e IM

Os 96 metabólitos plasmáticos foram utilizados como fatores de exposição, e os SNPs associados a esses 96 metabólitos plasmáticos foram selecionados como VIs. Uma análise de RM de duas amostras foi conduzida com I9_MI_STRICT como variável de desfecho. A análise identificou relações causais significativas (p<0,05) entre HLA DR em CDs e os seguintes metabólitos: níveis de 5-acetilamino-6-amino-3-metiluracila, razão glicina/alanina, razão serina/treonina, níveis de glicuronídeo de ácido desoxicólico, razão fosfato/N-acetilneuraminato, níveis de N-acetilglicina e níveis de glicosilceramida (d18:2/24:1, d18:1/24:2).

## Análise de RM de Mediação

Os efeitos de mediação (valores β) dos níveis de 5-acetilamino-6-amino-3-metiluracila, da razão glicina/alanina, da razão serina/treonina e dos níveis de N-acetilglicina na relação entre HLA DR em CDs e IM apresentaram sinais opostos aos efeitos totais, indicando que esses efeitos devem ser considerados como mascaradores.^12^ Portanto, os níveis de glicuronídeo de ácido desoxicólico, a razão fosfato/N-acetilneuraminato e a glicosilceramida foram selecionados para a análise de RM de mediação ( Tabela 1 , Tabela 2 ). Consequentemente, se pode inferir que os níveis de glicuronídeo de ácido desoxicólico podem atuar como um fator protetor contra IM, enquanto a razão fosfato/N-acetilneuraminato e a glicosilceramida podem atuar como fatores de risco para IM.

**Tabela 1:** – Resultados da análise de mediação por randomização mendeliana

Exposição	Resultado	Método Estatístico	OR	IC de 95%	Valor - p
Níveis de glucuronídeo de ácido desoxicólico	IM	VIP	0,94	0,89–0,99	0,013
Relação fosfato/N-acetilneuraminato	IM	VIP	1.06	1.01–1.11	0,017
Glicosilceramida	IM	VIP	1.08	1,00–1,16	0,044

*IM: infarto do miocárdio; VIP: variância inversa ponderada.*

**Tabela 2:** – Proporção dos efeitos de mediação mediados por metabólitos plasmáticos

Metabólito plasmático	Efeito Total (c)	Efeito das células imunes nos metabólitos plasmáticos (a)	Efeito dos metabólitos no IM (b)	Efeito mediado (ab)	Proporção mediada (ab/c)	Efeito Direto (c-ab)
Níveis de glucuronídeo de ácido deoxicólico	0,037	−0,040	−0,064	0,0026	0,070	0,034
Relação fosfato/ N-acetilneuraminato	0,037	0,029	0,058	0,0017	0,046	0,035
Glicosilceramida	0,037	0,031	0,078	0,0025	0,066	0,035

*IM: infarto do miocárdio.*

## Análise de genes e vias metabólicas associadas a células imunes e metabólitos

**Figura 7:**
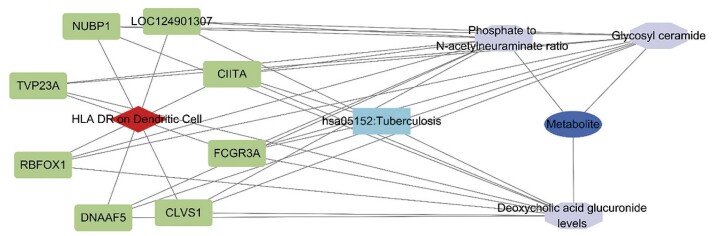
– Genes e vias metabólicas associadas a células imunes e metabólitos.

Foi realizada uma análise sistemática de SNPs associados ao HLA DR em CDs e três metabólitos: níveis de glicuronídeo de ácido desoxicólico, razão fosfato/N-acetilneuraminato e glicosilceramida. Posteriormente, uma rede de interação proteína-proteína foi construída utilizando o banco de dados STRING para explorar os potenciais mecanismos moleculares influenciados por essas variantes genéticas. A análise identificou oito genes-chave e uma via de sinalização que podem conectar esses fenótipos imunológicos e metabólitos, sugerindo seu potencial papel como mediadores entre as respostas imunes e a regulação metabólica (p<0,05) ( Figura 7 ). Essas descobertas fornecem uma base molecular para a compreensão dos mecanismos mais profundos dos fenótipos imunológicos e dos metabólitos no IM e estabelecem as bases para a subsequente validação funcional e exploração de potenciais alvos terapêuticos.

## Discussão

O antígeno leucocitário humano (HLA), também conhecido como complexo principal de histocompatibilidade (MHC), é codificado pelo complexo gênico HLA. O HLA pode ser classificado em antígenos de Classe I, Classe II e Classe III, sendo o HLA-DR um antígeno de Classe II. O HLA desempenha um papel crucial na resposta imune, na regulação imune e na vigilância imunológica, e está amplamente envolvido em áreas de pesquisa como doenças autoimunes, imunidade tumoral, transplante de órgãos e imunidade reprodutiva.^13^ O HLA-DR, como marcador de ativação, foi descrito inicialmente como um marcador de ativação de células T após exposição a diversos patógenos, antígenos ou em reações mistas de linfócitos. O HLA-DR aparece apenas na superfície de certos linfócitos T e, em comparação com outros marcadores de ativação (como CD69, CD25, CD71), aparece mais tarde durante o processo de ativação.^14^ As CDs desempenham um papel crucial na ligação entre os sistemas imunes inato e adaptativo. As CDs são células apresentadoras de antígenos (APCs) com uma capacidade única de ativar células T virgens. A disfunção das CDs pode levar a defeitos ou respostas imunes excessivas, que podem resultar em danos teciduais.^15^ Estudos relacionados demonstraram que^16^ tanto monócitos quanto CDs expressam HLA-DR. Em pacientes submetidos à cirurgia de revascularização do miocárdio, os perfis de expressão de moléculas coestimulatórias e de adesão em monócitos e CDs se alteram. A expressão de HLA-DR, moléculas coestimulatórias e moléculas de adesão em CDs e monócitos é suprimida e persiste no período pós-operatório. Essas alterações podem prejudicar os mecanismos de defesa do hospedeiro, afetando a migração celular, a apresentação de antígenos a células T virgens e a regulação de células T, aumentando, assim, o risco de infecções pós-operatórias. Uma diminuição na expressão de HLA-DR é frequentemente um marcador de imunossupressão e pode ser induzida por condições patológicas como sepse. Na sepse, a redução do HLA-DR reflete a supressão do sistema imunológico, enquanto na síndrome de liberação de citocinas, os monócitos são fortemente estimulados e a expressão de HLA-DR pode aumentar significativamente, refletindo uma resposta imune hiperativa.^17^ As moléculas de HLA-DR, características dos monócitos intermediários, são altamente expressas e desempenham um papel duplo nas respostas imunes. Elas podem tanto promover a inflamação quanto exibir efeitos anti-inflamatórios.^18^ A importância do HLA-DR na regulação imune é cada vez mais reconhecida, mas a pesquisa sobre seu papel no IM ainda é limitada. Estudos futuros precisam explorar o papel do HLA-DR na regulação imune durante o IM com maior profundidade e investigar estratégias terapêuticas mais precisas.

O glicuronídeo do ácido desoxicólico pode se ligar às células hospedeiras e participar da circulação secundária dos ácidos biliares, principalmente por meio de interações com receptores em células epiteliais intestinais, células imunes e outras células-alvo. Ao modular diretamente a proliferação, diferenciação e secreção de citocinas das células imunes, ele pode contribuir para a regulação das respostas imunes.^19^ Os ácidos biliares são sintetizados no fígado pela via clássica iniciada pela colesterol 7α-hidroxilase (CYP7A1) e têm sido propostos como potenciais biomarcadores para avaliar a gravidade de doenças cardiovasculares. Após a secreção dos ácidos biliares primários no intestino, eles são transformados por desconjugação bacteriana, 7α-desidroxilação e isomerização em vários ácidos biliares secundários, como o ácido litocólico, o ácido hiodesoxicólico e o ácido ursodesoxicólico. Esses ácidos biliares, como metabólitos da microbiota intestinal, têm demonstrado estreita associação com o desenvolvimento e a progressão da doença arterial coronariana.^20^ Do ponto de vista intervencionista, estratégias como a suplementação com probióticos e o transplante de microbiota fecal podem promover a produção de glicuronídeo de ácido desoxicólico, restaurando a homeostase da microbiota intestinal e, consequentemente, modulando o eixo de interação ácido biliar-microbiota e melhorando a disfunção vascular. Ao mesmo tempo, agentes farmacológicos já existentes podem influenciar essa via por meio de diferentes mecanismos: as estatinas podem alterar indiretamente a composição do pool de ácidos biliares; os sequestrantes de ácidos biliares podem modular a circulação entero-hepática; os agonistas de FXR/TGR5 podem regular diretamente a sinalização dos ácidos biliares; e os inibidores da β-glicuronidase podem ajudar a manter níveis estáveis de glicuronídeo de ácido desoxicólico, prevenindo a desconjugação microbiana dos ácidos biliares conjugados. Estudos demonstraram que o glicuronídeo de ácido desoxicólico pode regular a infiltração de macrófagos e influenciar a diferenciação de células Th17 por meio da via do receptor muscarínico de acetilcolina M2 (M2-mAChR)/Src, participando, assim, de respostas inflamatórias. Esses mecanismos podem estar intimamente relacionados à inflamação e à disfunção vascular no IM, com alterações nos níveis de glicuronídeo do ácido desoxicólico afetando potencialmente o recrutamento de células inflamatórias e o ambiente imunológico local durante a isquemia-reperfusão miocárdica, influenciando, em última análise, a extensão da lesão e do reparo miocárdico. Notavelmente, a pesquisa sobre o glicuronídeo do ácido desoxicólico no IM ainda é limitada. Dado o seu potencial terapêutico na doença arterial periférica, ele se apresenta como um promissor biomarcador e alvo terapêutico para o IM, oferecendo novas estratégias para a prevenção e o tratamento do IM e de doenças cardiovasculares.

O fosfato sérico está intimamente associado à calcificação arterial em diversas doenças humanas e animais, particularmente na hiperfosfatemia grave induzida por genes, que pode levar à calcificação generalizada. A calcificação causada pela hiperfosfatemia é considerada um importante mecanismo que contribui para o aumento da mortalidade em pacientes com doença renal crônica. Ao mesmo tempo, o papel do fosfato na calcificação da artéria coronária está primariamente relacionado à hiperfosfatemia. Mesmo quando os níveis séricos de fosfato permanecem dentro da faixa normal, um aumento nesses níveis é considerado um potencial fator de risco para morbidade e mortalidade cardiovascular na população em geral.^21 - 23^ O aumento da sobrecarga de fosfato promove, direta ou indiretamente, a calcificação da camada média das artérias e a hipertrofia ventricular esquerda, ambas aumentando a suscetibilidade dos pacientes à doença arterial coronariana. Os grânulos de fosfato de cálcio formados em condições de hiperfosfatemia promovem a transformação das células musculares lisas vasculares (CMLV) em células semelhantes a osteoblastos, fornecendo, assim, um arcabouço para a calcificação na camada média arterial. O aumento do fator de crescimento de fibroblastos 23 induzido pela sobrecarga excessiva de fosfato e a disrupção do metabolismo da vitamina D desempenham papéis significativos no desenvolvimento da hipertrofia miocárdica e da fibrose cardíaca.^24^O N-acetilneuraminato, também conhecido como ácido siálico, é uma molécula de açúcar natural comum, amplamente presente em muitas glicoproteínas e que desempenha um papel importante em sistemas biológicos. Ele ativa a via de sinalização Rho/ROCK (quinase de domínio espiralado associada a Rho) ao se ligar a RhoA e Cdc42, promovendo a migração de monócitos/macrófagos. O N-acetilneuraminato pertence à família dos monossacarídeos, com uma cadeia principal de seis carbonos e um alto grau de diversidade estrutural, o que o torna um potencial receptor para vírus. Está intimamente associado à transformação maligna, ao câncer e à metástase e invasão de grandes e pequenos vasos sanguíneos.^25^ Estudos relevantes demonstraram que^26^ níveis elevados de C16:0-glicosilceramida estão independentemente associados a um risco aumentado de morte cardíaca. Em um ensaio clínico randomizado e controlado, um ano de tratamento com liraglutida suprimiu significativamente as alterações nos níveis de C16:0-glicosilceramida no plasma de pacientes obesos, demonstrando uma redução em comparação ao grupo controle. Esses resultados sugerem que as glicosilceramidas, especialmente a C16:0-glicosilceramida, não apenas desempenham um papel importante na saúde cardiovascular, mas também podem servir como potenciais biomarcadores ou alvos terapêuticos. A liberação de ceramidas por meio da palmitoilação, juntamente com a subsequente resposta inflamatória e alterações no acoplamento elétrico, pode aumentar o risco de morte súbita cardíaca causada por taquicardia ventricular esquerda.^27^No entanto, existem poucos estudos sobre o papel da razão fosfato/N-acetilneuraminato e da glicosilceramida no IM. Portanto, este estudo visa fornecer informações metabólicas valiosas e revelar a conexão entre anormalidades metabólicas e respostas inflamatórias em doenças cardiovasculares. No futuro, ao explorarmos mais a fundo os papéis potenciais desses metabólitos no IM e em outras doenças cardiovasculares, poderemos oferecer novas direções de pesquisa para o diagnóstico precoce, terapia direcionada e o desenvolvimento de estratégias médicas personalizadas

Nubp1 é um componente essencial do complexo de suporte do cluster ferro-enxofre citosólico, envolvido na montagem e no transporte de clusters ferro-enxofre. Sua regulação positiva compensatória pode conferir efeitos protetores no miocárdio isquêmico; no entanto, é insuficiente para neutralizar completamente os defeitos na fosforilação oxidativa (OXPHOS) e, portanto, pode estar associada à lesão miocárdica e à progressão do IM.^28^ TVP23A codifica a proteína homóloga A da vesícula da rede trans-Golgi 23 (anteriormente FAM18A), que está primariamente envolvida nas funções de transporte e secreção das vesículas de Golgi. Ela é altamente expressa em macrófagos e plasmócitos, sugerindo um papel potencial na secreção de células imunes e em respostas inflamatórias.^29^ A alta expressão de TVP23A nessas células pode influenciar a liberação de mediadores inflamatórios e a resposta ao estresse miocárdico, contribuindo assim para os processos patológicos do IM. Rbfox1 é um fator de splicing que se liga ao RNA e regula o splicing alternativo do éxon do gene CaV1.2 (Cacna1c). Ele é altamente expresso tanto no desenvolvimento neural quanto nas CMLV, afetando a contração do músculo liso vascular e a regulação do tônus vascular.^30^ Dessa forma, pode contribuir para a ocorrência, progressão e remodelamento patológico da isquemia miocárdica e do IM, alterando a hemodinâmica coronária, a complacência vascular e a função endotelial local.^31^ DNAAF5 pertence à família de proteínas com repetições HEAT (incluindo huntingtina, fator de elongação eucariótico 3, PP2A, mTOR, etc.), caracterizada por motivos de repetição α-helicoidal de aproximadamente 30 a 40 aminoácidos. DNAAF5 é um membro da família DNAAF, que compreende pelo menos 11 proteínas citoplasmáticas. Mutações homozigóticas com perda de função resultam em letalidade embrionária, enquanto mutações heterozigóticas compostas (mutação de sentido trocado mais deleção) causam distúrbios graves, como hidrocefalia e morte precoce.^32^ Atualmente, o papel específico do DNAAF5 no IM permanece incerto. A CLVS1 é uma proteína chave na endocitose mediada por clatrina e regula o transporte de α-tocoferol e os níveis de estresse oxidativo intracelular por meio de sua interação com a proteína de transferência de α-tocoferol (αTTP), levando ao acúmulo de espécies reativas de oxigênio (EROs).^33^ O excesso de EROs não apenas aumenta o tamanho do infarto e prejudica a função miocárdica, mas também promove a remodelação do ventrículo esquerdo, com seus níveis correlacionando-se estreitamente com a gravidade do IM. O gene FCGR3A, localizado no cromossomo 1, codifica o receptor de baixa afinidade FcγRIIIa/CD16a, que é expresso principalmente em células natural killer, macrófagos e mastócitos, e participa da citotoxicidade celular dependente de anticorpos (ADCC). Seu polimorfismo funcional comum, F158V, resulta de uma variação de um único nucleotídeo que leva à presença de fenilalanina (F) ou valina (V) nessa posição, e apresenta diferenças significativas na distribuição genotípica em pacientes com aterosclerose ou acidente vascular cerebral isquêmico. Como a aterosclerose é uma base patológica fundamental para a ocorrência e progressão do IM, esse polimorfismo genético também pode estar intimamente associado à suscetibilidade ao IM. CIITA é um gene regulador transcricional crítico no genoma humano, responsável principalmente pelo controle da expressão dos genes do MHC classe II. As moléculas MHC II são expressas principalmente em APCs, como CDs, macrófagos e células B, e têm a função de apresentar antígenos exógenos às células T CD4⁺, iniciando assim respostas imunes adaptativas.^34 , 35^ A CIITA promove respostas inflamatórias ao aumentar a atividade das células B e das células T CD4⁺; em condições como a hiper-homocisteinemia, a resposta imune-inflamatória amplificada pela via PKM2-CREB1-CIITA tem sido implicada.^36^ Mecanismos semelhantes podem operar na íntima coronária e em regiões miocárdicas isquêmicas, exacerbando a inflamação local e promovendo a instabilidade da placa, aumentando assim o risco de IM. O LOC124901307 pertence à categoria de RNA não codificante (ncRNA); embora sua função específica permaneça obscura e estudos sejam escassos, ele pode contribuir para a ocorrência e progressão do IM modulando a expressão gênica ou regulando vias relacionadas à homeostase vascular. Indivíduos com histórico de tuberculose apresentam risco significativamente aumentado de doenças cardiovasculares, incluindo IM, síndrome coronariana aguda, acidente vascular cerebral isquêmico e doença arterial periférica.^37^ Isso pode estar relacionado à resposta inflamatória crônica, à ativação imunológica e à disfunção endotelial induzidas pela infecção por tuberculose. Estudos futuros poderão elucidar ainda mais seus mecanismos moleculares e potencial clínico, fornecendo novas estratégias para o diagnóstico precoce e intervenção direcionada no IM.

Embora este estudo forneça novas perspectivas para a compreensão do IM, ele ainda apresenta algumas limitações. Primeiro, ele se baseia em dados de GWAS disponíveis publicamente para a análise de RM, que podem ser influenciados por fatores como qualidade dos dados, viés de seleção da amostra e heterogeneidade genética. Segundo, os dados utilizados neste estudo são derivados principalmente de populações europeias, e os dados de GWAS para células imunes e metabólitos provêm, em sua maioria, de populações com origens genéticas relativamente homogêneas e características específicas. Essa homogeneidade pode limitar a validade externa das descobertas, visto que existem diferenças na arquitetura genética, na composição da microbiota intestinal, nos hábitos alimentares e no estilo de vida entre as populações. Essas diferenças podem afetar as respostas imunes, os níveis de metabólitos e a suscetibilidade ao IM, potencialmente levando a relações causais distintas em populações não europeias. Além disso, devido à falta de informações e relatos sobre metabólitos desconhecidos, este estudo enfrenta desafios na interpretação e discussão dos resultados da análise de RM. Pesquisas futuras devem coletar mais dados individuais de pacientes com IM para validar e ampliar as descobertas, incorporando populações mais diversas e considerando a interação de múltiplos fatores para elucidar de forma mais abrangente a patogênese do IM.

## Conclusão

Neste estudo, a análise de RM de mediação foi empregada para avaliar sistematicamente as relações causais e os efeitos de mediação entre 731 características de células imunes, 1.400 metabólitos plasmáticos e IM. Um total de 38 fenótipos de células imunes e 96 metabólitos plasmáticos foram identificados como tendo associações causais com o IM. Os resultados demonstraram uma relação causal positiva significativa entre o HLA-DR em CDs e o IM, enquanto a análise de RM reversa não revelou nenhum efeito do IM sobre o HLA-DR em CDs. Análises adicionais de RM de mediação indicaram que o HLA-DR em CDs pode influenciar o desenvolvimento do IM por meio dos níveis de glicuronídeo de ácido desoxicólico, da razão fosfato/N-acetilneuraminato e da glicosilceramida, com proporções de efeitos de mediação de 7%, 4,6% e 6,6%, respectivamente. Níveis elevados de HLA-DR em CDs podem aumentar o risco de IM ao diminuir os níveis de glicuronídeo de ácido desoxicólico, aumentar a razão fosfato/N-acetilneuraminato e elevar os níveis de glicosilceramida. Por meio de análise sistemática de SNPs associados ao HLA-DR em CDs e a esses três metabólitos, identificamos genes-chave, incluindo NUBP1, TVP23A, RBFOX1, DNAAF5, CLVS1, FCGR3A, CIITA, LOC124901307, bem como a via de sinalização da tuberculose, que podem mediar potenciais interações entre fenótipos imunológicos e características metabólicas ( Ilustração Central ). Em resumo, este estudo fornece novas perspectivas sobre a patogênese do IM, oferece evidências adicionais para elucidar seus mecanismos subjacentes e pode servir como referência para a identificação de potenciais alvos terapêuticos.
