# Supplementary material for: Associação Causal entre Células Imunes Mediadas por Metabólitos Plasmáticos e Infarto do Miocárdio: Um Estudo de Randomização Mendeliana
Source: Arq Bras Cardiol. 2026 Apr 28;123(4):e20260026. [Article in Portuguese] doi: 10.36660/abc.20250026 (PMC13155783; doi:10.36660/abc.20250026)
Supplement: Translation

# Translation: The Causal Association between Plasma Metabolites-Mediated Immune Cells and Myocardial Infarction: A Mendelian Randomization Study

**DOI:** 10.36660/abc.20250026i

**Authors:** Yue Liu, Xing Chen, Yingzhi Liu, Yvwen Lu, Yun Zhu, Lingli Chen, Jie Li

**Affiliations:** 1 China Hunan University of Chinese Medicin, Changsha, Hunan – China

**Keywords:** Mendelian Randomization Analysis, Biomarkers, Myocardial Infarction

## Abstract

**Background:**

blaMyocardial infarction (MI), as an acute cardiovascular event, has been shown in previous studies to be associated with immune responses and metabolites. However, the causal relationship between immune cells and MI, as well as the mediating role of plasma metabolites, has not been clearly confirmed.

**Objectives:**

Systematic analysis of the causal association between 731 immune cells and MI, as well as the mediating roles of 1400 plasma metabolites.

**Methods:**

Bidirectional Mendelian randomization (MR) analysis was used to explore the causal relationship between immune cells and MI, and one-step and two-step mediation analyses were performed to identify plasma metabolites. The analysis methods included inverse variance weighted (p<0.05), weighted median (p<0.05), simple mode (p<0.05), weighted mode (p<0.05), MR-Egger regression (p<0.05), MR-Egger intercept (p>0.05) and MR-PRESSO (p>0.05) to assess the reliability of the results. SNPs associated with immune cells and metabolites were subjected to gene selection using the STRING database, followed by enrichment analysis.

**Results:**

A total of 44 immune cells were identified to have a causal association with MI (p<0.05), among which HLA DR on Dendritic Cell (ebi-a-GCST90002106) showed the strongest significance (p<0.001). A total of 19 SNPs were identified (OR=1.038, 95% CI 1.018-1.059). Reverse analysis showed no statistical significance (p>0.05). Plasma metabolites, including Deoxycholic acid glucuronide levels, Phosphate to N-acetylneuraminate ratio, and Glycosyl ceramide, played a mediating role in immune cells and MI, with mediation effects of 7.0%, 4.6%, and 6.6%, respectively. STRING database analysis identified eight key genes and one signaling pathway that potentially link the immune phenotypes and the three metabolites (p< 0.05).

**Conclusions:**

This study identified a causal relationship between immune cells and MI. The metabolites Deoxycholic acid glucuronide levels, Phosphate to N-acetylneuraminate ratio, and Glycosyl ceramide played a partial mediating role in this causal relationship.

## Introduction

Cardiovascular disease is a leading cause of death worldwide, with ischemic heart disease accounting for 49.2% of these deaths. Myocardial infarction (MI) is typically caused by the obstruction of arteries or bypass grafts by thrombosis, characterized by a sudden reduction in blood flow to the myocardium, ultimately leading to heart failure and myocardial death. Despite significant progress in MI treatment, it remains a globally prevalent disease with high morbidity and mortality. Although treatment methods have improved, many challenges remain in identifying an ideal therapeutic strategy.^1^During MI, myocardial cell death occurs, the tissue in the infarcted area undergoes necrosis, and an inflammatory response is activated. Immune cells can both promote myocardial cell death and inflammation, as well as facilitate the regeneration of damaged myocardium, making them an important component in disease research.^2^Myocardial remodeling after MI is a complex repair process characterized by the infiltration of various types of immune cells.^3^Related metabolic diseases, such as hypercholesterolemia, hypertriglyceridemia, type 2 diabetes, and hypertension, are associated with the activation of immune cells. These factors may interfere with the inflammatory healing process after MI. It is evident that there is a complex interaction between immune cells and metabolites, and this intricate relationship may jointly contribute to the occurrence and progression of MI. This also suggests that metabolites could serve as mediators influencing immune cells and disease development. Exploring the mediating role of plasma metabolites in the relationship between immune cells and MI may deepen the overall understanding of MI and provide new therapeutic targets and research directions.

Mendelian randomization (MR) analysis is a statistical technique that uses genetic variations as instrumental variables (IVs) to explore causal relationships between exposure factors and outcomes. This method effectively utilizes the results of genome-wide association studies (GWAS), using genetic variations as IVs to investigate the causal relationship between exposure and outcome.^4^Currently, there is a lack of research on the causal relationship between plasma metabolites-mediated immune cells and MI. This study aims to use the MR method to explore the causal relationship between immune cells and MI, analyze the correlation between relevant metabolites and MI, and reveal how immune cells influence the onset and progression of MI through specific plasma metabolite pathways. The findings of this study are expected to provide theoretical support for clinical treatment strategies based on metabolite regulation, promote the development of new therapeutic approaches, and ultimately improve the prognosis of MI patients.

## Materials and methods

### Study design

This study adopts a two-step mediation MR analysis to evaluate the role of plasma metabolites in immune cell regulation of MI. First, through a two-sample bidirectional MR analysis, immune cells are treated as exposure factors and MI as the outcome variable to assess the causal relationship between 731 immune cells and MI, and to derive the total effect (beta_all). At the same time, when MI is used as the exposure factor, and the 731 immune cells as the outcome variable, a reverse MR analysis is performed to ensure that no causal relationship is found. Secondly, 47 immune cells related to MI are selected. Through a two-sample MR analysis, the causal relationship between the most significantly associated immune cells and 1400 plasma metabolites is analyzed to derive the effect size (beta1) of the mediating metabolites. Subsequently, the selected plasma metabolites are treated as exposure factors and MI as the outcome variable to further assess the causal relationship between them and derive beta2. By combining beta_all, beta1, and beta2, mediation analysis is performed to evaluate the mediating role of plasma metabolites in immune cell regulation of MI, systematically assessing their impact. Finally, we systematically integrated and analyzed the SNPs of immune cells and metabolites, and employed the STRING database to identify the related genes. Subsequently, enrichment analysis was performed to screen the involved pathways, and an interaction network was constructed to identify key nodes and potential pathways. This approach comprehensively reveals the potential interactive mechanisms between immune cells and metabolites in the development and progression of diseases, and provides a theoretical basis and direction for subsequent intervention studies.

### Data sources

The data for this study were sourced from the GWAS summary statistics of 731 immune cell (GCST90001391-GCST90002121) phenotypes available in the GWAS Catalog.^5^Among the 731 immune cells, data were obtained through flow cytometry analysis. Of these, 118 represent absolute cell counts (AC), 389 reflect median fluorescence intensity (MFI), 32 are morphological parameters, and 192 represent relative cell. Specifically, these cells were carefully classified into seven major groups, including B cells, classical dendritic cells, mature T cells, monocytes, myeloid-derived cells, TBNK lymphocytes (T cells, B cells, natural killer cells), and regulatory T cells (Treg). The original GWAS data with immune characteristics were derived from 3757 unrelated individuals from the Sardinian population, with no overlap between cohorts. Approximately 22 million high-density SNP genotypes were input, and after adjusting for confounding factors (such as sex, age, etc.), the correlation analysis was performed based on the Sardinian reference panel.^6^The GWAS data on circulating metabolites used in this study included information from 8,299 individuals and covered a total of 1,400 metabolites, comprising 1,091 metabolites and 309 metabolite ratios, of which 850 were identified as known metabolites. These data were derived from the Canadian Longitudinal Study on Aging (CLSA) cohort, which represents an important resource for metabolomics GWAS and provides valuable information for investigating metabolic mechanisms and their associations with genetic variation. All data are publicly available, with accession numbers ranging from GCST90199621 to GCST90201020. The MI data for this study were sourced from the FinnGen database, with data ID number I9_MI_STRICT. This database contains large-scale genomic data from Finland, which has undergone rigorous screening and quality control to ensure the representativeness of the samples and the accuracy of the data. The total sample size is 406,565 individuals, including 378,019 controls and 28,546 disease cases. The data collection covers individuals of different ages, sexes, and clinical backgrounds. As this study uses publicly available summary data previously published, no additional ethical approval is required. All original data were obtained with informed consent from the participants. All data used in this study were obtained from publicly available GWAS or FinnGen summary statistics. No individual-level samples were accessed or retained in this study. Therefore, the sample size was determined by all available data in the original GWAS or FinnGen datasets.

### Selection of instrumental variables

SNPs are used as a powerful tool to assess potential associations. These SNP data are typically sourced from GWAS and are considered as IVs.^7^In this study, SNPs are used as IVs to assess causal relationships. The MR study design needs to satisfy three basic assumptions: (1) IVs are strongly associated with the exposure, (2) IVs are independent of any confounding factors. (3) IVs affect the outcome solely through the exposure.^8^Considering that using the traditional significance threshold of $P<5\times 10^{-8}$ may limit the number of available SNPs in the study, thereby affecting the feasibility of the analysis, this study adopted a threshold of $P<1\times 10^{-5}$ to increase the number of SNPs available for analysis. This approach effectively enhances the number of usable SNPs, thereby increasing the statistical power of the analysis and ensuring the reliability and comprehensiveness of the results. To remove linkage disequilibrium (LD), a threshold of (kb=10,000, r^2^=0.001) was applied. The strength of the IVs was evaluated using the F-statistic formula: $F=\left[R^{2}\times (N-1-K)\right]/\left[K\times \left(1-R^{2}\right)\right]$ , where R^2^represents the proportion of variance in the exposure explained by genetic variation, N represents the sample size of the exposure GWAS, and K represents the number of SNPs. If the corresponding F-statistic is >10, it indicates no significant weak instrument bias, thus increasing the reliability of the MR analysis results.^9^

### Statistical methods

All statistical analyses and plotting were performed using R4.4.0 and RStudio software, with the “TwosampleMR” package and others used for MR and mediation analyses. A p-value of <0.05 was considered statistically significant. This study employed five different methods to assess bidirectional causal effects, including inverse variance weighted (IVW), weighted median (WM), simple mode, weighted mode, and MR-Egger regression. Since IVW is considered robust in causal inference, it was chosen as the primary method for estimating causal effects. To address potential issues such as pleiotropy, MR-Egger regression analysis and MR-PRESSO validation were performed. Heterogeneity was assessed using Cochran’s Q statistic, sensitivity analysis was conducted using leave-one-out analysis, and potential biases were evaluated through funnel plots, particularly to check for pleiotropy and publication bias. We used Odds Ratio (OR) and the corresponding 95% Confidence Interval (CI) to accurately quantify and describe the magnitude of the effect size and its uncertainty. The OR, as a statistical measure, provides a clear reflection of the relative probability of an event occurring between two or more groups, while the 95% CI offers the reliability range of the OR estimate, further enhancing the interpretability and credibility of the study results.

### Mediation analysis

A two-step MR design was used for mediation analysis to investigate whether plasma metabolites mediate the causal pathway between immune cells and MI. First, to assess the causal relationship between immune cells and MI, a two-sample MR analysis was applied. The purpose of this step was to verify the association between immune cells and MI and calculate the overall effect (c). Then, a reverse MR analysis was conducted, with MI as the exposure factor and immune cells as the outcome variable, to further examine whether a causal relationship exists between immune cells and MI.

We analyzed whether plasma metabolites mediate the relationship between immune cells and MI outcomes, employing a two-stage MR design to further explore this mediating effect. The mediation MR analysis includes effect a, effect b, and the mediating effect (ab), with the direct effect ( $c^{\prime }=c-a\times b$ ) calculated. The direct effect refers to the influence of immune cells on MI, while the mediating effect reflects the impact of immune cells on MI through the mediation of plasma metabolites. Finally, by comparing the indirect effect and total effect, we calculated the percentage of the mediating effect ( Figure 1 ).^10 , 11^

**Figure 1:** – (A) Overall effect between immune cells and myocardial infarction (MI): c represents the overall effect using immune cells as the exposure and MI as the outcome, while d represents the total effect using MI as the exposure and immune cells as the outcome. (B) Decomposition of the overall effect into: (1) Mediation effect (ab) using the two-step method, where a represents the effect of immune cells on metabolites, and b represents the effect of metabolites on MI. (2) Direct effect ( $c^{\prime }=c-a\times b$ ).

### MR analysis results

#### Causal relationship between 731 immune cells and MI

In this study, to explore the causal relationship between immune cells and MI, 731 immune cell phenotypes were used as exposure factors, and MI as the outcome variable, with 731 forward two-sample MR analyses conducted after removing LD and weak IVs. The results revealed that 47 immune cell phenotypes were causally associated with MI. Sensitivity analysis identified 9 immune cell phenotypes with heterogeneity and horizontal pleiotropy, and these 9 phenotypes were removed. After removal, IVW analysis showed that 38 immune cell phenotypes had reliable causal relationships with MI ( Figure 2 ). Among these, the immune cell phenotype HLA DR on Dendritic Cell (ebi-a-GCST90002106) was the most significant MI risk factor. The causal relationship between HLA DR on Dendritic Cells and MI showed no heterogeneity or horizontal pleiotropy, as indicated by Cochran’s Q test, MR-PRESSO test, and MR-Egger intercept test.

**Figure 2:** – MR Results of Immune Cells and MI (IVW Method).

The reverse MR analysis results of HLA DR on Dendritic Cell and MI (IVW method, p>0.05) showed no statistical significance, indicating that there is no reverse causal relationship between plasma metabolites and MI. The MR sensitivity analysis, forest plot, and scatter plot for HLA DR on Dendritic Cell and MI are shown in Figures 3 , 4 , and 5 .

**Figure 3:** – MR Sensitivity Analysis Results of HLA DR on Dendritic Cell and MI.

**Figure 4:** – Forest Plot Results of MR Analysis Between HLA DR on Dendritic Cell and MI.

**Figure 5:** – Scatter Plot Results of MR Analysis Between HLA DR on Dendritic Cell and MI.

## MR analysis of 1400 plasma metabolites and immune cells

To reveal the mechanisms through which the selected immune cells influence metabolic phenotypes and their impact on MI, we conducted MR analysis between HLA DR on Dendritic Cell and 1400 plasma metabolite phenotypes using five methods: MR Egger, Weighted Median, IVW, Simple Mode, and Weighted Mode. The IVW method was used as the primary testing method. After analysis, 96 plasma metabolites were identified, and sensitivity analysis revealed no heterogeneity or horizontal pleiotropy ( Figure 6 ).

**Figure 6:** – Analysis results of HLA DR on Dendritic Cells with 1,400 plasma metabolites.

## MR analysis of 96 plasma metabolites and MI

The 96 plasma metabolites were used as exposure factors, and SNPs associated with these 96 plasma metabolites were selected as IVs. Two-sample MR analysis was conducted with I9_MI_STRICT as the outcome variable. The analysis identified significant causal relationships (p<0.05) between HLA DR on Dendritic Cell and the following metabolites: 5-acetylamino-6-amino-3-methyluracil levels, Glycine to alanine ratio, Serine to threonine ratio, Deoxycholic acid glucuronide levels, Phosphate to N-acetylneuraminate ratio, N-acetylglycine levels, and Glycosyl ceramide (d18:2/24:1, d18:1/24:2) levels.

## Mediation MR analysis

The mediation effects (β values) of 5-acetylamino-6-amino-3-methyluracil levels, Glycine to alanine ratio, Serine to threonine ratio, and N-acetylglycine levels on the relationship between HLA DR on Dendritic Cell and MI were found to have opposite signs to the total effects, indicating that these effects should be considered as masking effects.^12^Therefore, Deoxycholic acid glucuronide levels, Phosphate to N-acetylneuraminate ratio, and Glycosyl ceramide were selected for mediation MR analysis ( Table 1 , Table 2 ). Accordingly, it can be inferred that Deoxycholic acid glucuronide levels may serve as a protective factor for MI, whereas Phosphate to N-acetylneuraminate ratio and Glycosyl ceramide may act as risk factors for MI.

**Table 1:** – Results of mediation MR analysis

Exposure	Outcome	Statistical Method	OR	95%CI	p-value
Deoxycholic acid glucuronide levels	MI	IVW	0.94	0.89–0.99	0.013
Phosphate to N-acetylneuraminate ratio	MI	IVW	1.06	1.01–1.11	0.017
Glycosyl ceramide	MI	IVW	1.08	1.00–1.16	0.044

*MI: myocardial infarction; IVW: inverse variance weighted.*

**Table 2:** – Proportion of mediation effects mediated by plasma metabolites

Plasma Metabolite	Total Effect (c)	Effect of Immune Cells on Plasma Metabolites(a)	Effect of Metabolites on MI (b)	Mediated Effect (ab)	Proportion Mediated (ab/c)	Direct Effect (c-ab)
Deoxycholic acid glucuronide levels	0.037	−0.040	−0.064	0.0026	0.070	0.034
Phosphate to N-acetylneuraminate ratio	0.037	0.029	0.058	0.0017	0.046	0.035
Glycosyl ceramide	0.037	0.031	0.078	0.0025	0.066	0.035

*MI: myocardial infarction.*

## Analysis of genes and pathways associated with immune cells and metabolites

A systematic analysis was performed on SNPs associated with HLA DR on Dendritic Cells and three metabolites: Deoxycholic acid glucuronide levels, Phosphate to N-acetylneuraminate ratio, and Glycosyl ceramide. Subsequently, a protein–protein interaction network was constructed using the STRING database to explore the potential molecular mechanisms influenced by these genetic variants. The analysis identified eight key genes and one signaling pathway that may link these immune phenotypes and metabolites, suggesting their potential role as mediators between immune responses and metabolic regulation (p<0.05) ( Figure 7 ). These findings provide a molecular basis for understanding the deeper mechanisms of immune phenotypes and metabolites in MI, and lay the groundwork for subsequent functional validation and exploration of potential therapeutic targets.

**Figure 7:** – Genes and Pathways Associated with Immune Cells and Metabolites.

## Discussion

Human leukocyte antigen (HLA), also known as the major histocompatibility complex (MHC), is encoded by the HLA gene complex. HLA can be classified into Class I antigens, Class II antigens, and Class III antigens, with HLA-DR being a Class II antigen. HLA plays a crucial role in immune response, immune regulation, and immune surveillance, and is widely involved in research areas such as autoimmune diseases, tumor immunity, organ transplantation, and reproductive immunity.^13^HLA-DR, as an activation marker, was first described as a marker of T cell activation after exposure to various pathogens, antigens, or in mixed lymphocyte reactions. HLA-DR appears only on the surface of certain T lymphocytes and, compared to other activation markers (such as CD69, CD25, CD71), it appears later during the activation process.^14^Dendritic cells (DCs) play a crucial role in bridging the innate and adaptive immune systems. DCs are antigen-presenting cells with a unique ability to activate naive T cells. Dysfunction of DCs can lead to defects or excessive immune responses, which may result in tissue damage.^15^Related studies have shown that^16^monocytes and dendritic cells (DCs) both express HLA-DR. In patients undergoing coronary artery bypass grafting (CABG), the expression profiles of costimulatory molecules and adhesion molecules on monocytes and DCs change. The expression of HLA-DR, costimulatory molecules, and adhesion molecules on both DCs and monocytes is suppressed and persists in the postoperative period. These changes may impair the host’s defense mechanisms, affecting cell migration, antigen presentation to naïve T cells, and T cell regulation, thereby increasing the risk of postoperative infections. A decrease in HLA-DR is often a marker of immunosuppression and can be induced by pathological conditions such as sepsis. In sepsis, the reduction in HLA-DR reflects immune system suppression, while in cytokine release syndrome (CRS), monocytes are strongly stimulated, and HLA-DR expression may significantly increase, reflecting an overactive immune response.^17^HLA-DR molecules, as a characteristic of intermediate monocytes, are highly expressed and play a dual role in immune responses. They can both promote inflammation and exhibit anti-inflammatory effects.^18^The importance of HLA-DR in immune regulation is increasingly recognized, but research on its role in MI is still limited. Future studies need to explore the role of HLA-DR in immune regulation during MI in greater depth and investigate more precise therapeutic strategies.

Deoxycholic acid glucuronide can bind to host cells and participate in the secondary bile acid circulation, primarily through interactions with receptors on intestinal epithelial cells, immune cells, and other target cells. By directly modulating the proliferation, differentiation, and cytokine secretion of immune cells, it may contribute to the regulation of immune responses.^19^Bile acids are synthesized in the liver via the classical pathway initiated by cholesterol 7α-hydroxylase (CYP7A1) and have been proposed as potential biomarkers for assessing the severity of cardiovascular diseases. After primary bile acids are secreted into the intestine, they are transformed by bacterial deconjugation, 7α-dehydroxylation, and isomerization into various secondary bile acids, such as lithocholic acid (LCA), hyodeoxycholic acid (HDCA), and ursodeoxycholic acid (UDCA). These bile acids, as metabolites of the gut microbiota, have been shown to be closely associated with the development and progression of coronary artery disease.^20^From an interventional perspective, strategies such as probiotic supplementation and fecal microbiota transplantation may promote the production of deoxycholic acid glucuronide by restoring gut microbial homeostasis, thereby modulating the bile acid–microbiota interaction axis and improving vascular dysfunction. Meanwhile, existing pharmacological agents may influence this pathway through different mechanisms: statins can indirectly alter the composition of the bile acid pool; bile acid sequestrants can modulate the enterohepatic circulation; FXR/TGR5 agonists can directly regulate bile acid signaling; and β-glucuronidase inhibitors may help maintain stable levels of deoxycholic acid glucuronide by preventing microbial deconjugation of conjugated bile acids. Studies have shown that deoxycholic acid glucuronide can regulate macrophage infiltration and influence Th17 cell differentiation via the M2 muscarinic acetylcholine receptor (M2-mAChR)/Src pathway, thereby participating in inflammatory responses. These mechanisms may be closely related to MI inflammation and vascular dysfunction, with alterations in deoxycholic acid glucuronide levels potentially affecting the recruitment of inflammatory cells and the local immune environment during myocardial ischemia-reperfusion, ultimately influencing the extent of myocardial injury and repair. Notably, research on deoxycholic acid glucuronide in MI remains limited. Given its potential therapeutic value in peripheral arterial disease, it holds promise as a biomarker and therapeutic target for MI, offering new strategies for the prevention and treatment of MI and cardiovascular diseases.

Serum phosphate is closely associated with arterial calcification in various human and animal diseases, particularly in gene-induced severe hyperphosphatemia, which can lead to widespread calcification. Calcification caused by hyperphosphatemia is considered an important mechanism contributing to increased mortality in patients with chronic kidney disease (CKD). At the same time, the role of phosphate in coronary artery calcification (CAC) is primarily related to hyperphosphatemia. Even when serum phosphate levels remain within the normal range, an increase in phosphate levels is considered a potential risk factor for cardiovascular morbidity and mortality in the general population.^21 - 23^Increased phosphate loading directly or indirectly promotes medial arterial calcification and left ventricular hypertrophy, both of which increase the susceptibility of patients to coronary artery disease. Calcium-phosphate granules formed under hyperphosphatemia conditions promote the transformation of vascular smooth muscle cells (VSMCs) into osteoblast-like cells, thereby providing a scaffold for calcification in the arterial media. The increased fibroblast growth factor 23 induced by excessive phosphate load and the disruption of vitamin D metabolism play significant roles in the development of myocardial hypertrophy and cardiac fibrosis.^24^N-acetylneuraminate, also known as sialic acid, is a common natural sugar molecule widely present in many glycoproteins and plays an important role in biological systems. It activates the Rho/Rho-associated coiled-coil kinase (ROCK) signaling pathway by binding with Rho A and Cdc42, promoting the migration of monocytes/macrophages. N-acetylneuraminate belongs to the monosaccharide family, with a six-carbon backbone and a high degree of structural diversity, making it a potential receptor for viruses. It is closely associated with malignant transformation, cancer, and the metastasis and invasion of large and small vessel complications.^25^Relevant studies have shown that^26^high levels of C16:0-glycosylceramide are independently associated with an increased risk of cardiac death. In a randomized controlled clinical trial, one year of liraglutide treatment significantly suppressed changes in C16:0-glycosylceramide levels in the plasma of obese patients, showing a reduction compared to the control group. These results suggest that glycosylceramides, especially C16:0-glycosylceramide, not only play an important role in cardiovascular health but may also serve as potential biomarkers or therapeutic targets. The release of ceramides through palmitoylation, along with the subsequent inflammatory response and changes in electrical coupling, may increase the risk of sudden cardiac death (SCD) caused by left ventricular ventricular tachycardia.^27^However, there is limited research on the role of the Phosphate to N-acetylneuraminate ratio and Glycosyl ceramide in MI. Therefore, this study aims to provide valuable metabolic information and reveal the connection between metabolic abnormalities and inflammatory responses in cardiovascular diseases. In the future, by further exploring the potential roles of these metabolites in MI and other cardiovascular diseases, we may offer new research directions for early diagnosis, targeted therapy, and the development of personalized medical strategies.

Nubp1 is an essential component of the cytosolic iron-sulfur cluster (ISC) scaffold complex, involved in the assembly and transport of iron-sulfur clusters. Its compensatory upregulation may confer protective effects on ischemic myocardium; however, it is insufficient to fully counteract defects in oxidative phosphorylation (OXPHOS), and thus may be associated with myocardial injury and the progression of MI.^28^TVP23A encodes the trans-Golgi network vesicle protein 23 homolog A (formerly FAM18A), which is primarily involved in the transport and secretory functions of Golgi vesicles. It is highly expressed in macrophages and plasma cells, suggesting a potential role in immune cell secretion and inflammatory responses.^29^The high expression of TVP23A in these cells may influence the release of inflammatory mediators and the myocardial stress response, thereby contributing to the pathological processes of MI. Rbfox1 is an RNA-binding splicing factor that regulates alternative exon splicing of the CaV1.2 (Cacna1c) gene. It is highly expressed in both neural development and vascular smooth muscle cells, affecting vascular smooth muscle contraction and the regulation of vascular tone.^30^It may thereby contribute to the occurrence, progression, and pathological remodeling of myocardial ischemia and MI by altering coronary hemodynamics, vascular compliance, and local endothelial function.^31^DNAAF5 belongs to the HEAT-repeat protein family (including Huntingtin, eukaryotic elongation factor 3, PP2A, mTOR, etc.), characterized by α-helical repeat motifs of approximately 30–40 amino acids. DNAAF5 is a member of the DNAAF family, which comprises at least 11 cytoplasmic proteins. Homozygous loss-of-function mutations result in embryonic lethality, whereas compound heterozygous mutations (missense plus deletion) cause severe disorders such as hydrocephalus and early death.^32^Currently, the specific role of DNAAF5 in MI remains unclear. CLVS1 is a key protein in clathrin-mediated endocytosis (CME) and regulates the transport of α-tocopherol and intracellular oxidative stress levels through its interaction with α-tocopherol transfer protein (αTTP), leading to the accumulation of reactive oxygen species (ROS).^33^Excessive ROS not only enlarges the infarct size and impairs myocardial function but also promotes left ventricular remodeling, with its levels closely correlating with the severity of MI. The FCGR3A gene, located on chromosome 1, encodes the low-affinity receptor FcγRIIIa/CD16a, which is primarily expressed in natural killer cells, macrophages, and mast cells, and participates in antibody-dependent cell-mediated cytotoxicity (ADCC). Its common functional polymorphism, F158V, results from a single nucleotide variation leading to either phenylalanine (F) or valine (V) at this position, and exhibits significant genotype distribution differences in patients with atherosclerosis or ischemic stroke. Since atherosclerosis is a key pathological basis for the occurrence and progression of MI, this gene polymorphism may also be closely associated with MI susceptibility. CIITA is a critical transcriptional regulatory gene in the human genome, primarily responsible for controlling the expression of MHC class II genes. MHC II molecules are mainly expressed on antigen-presenting cells (APCs), such as dendritic cells, macrophages, and B cells, and function to present exogenous antigens to CD4⁺ T cells, thereby initiating adaptive immune responses.^34 , 35^CIITA promotes inflammatory responses by enhancing the activity of B cells and CD4⁺ T cells; under conditions such as hyperhomocysteinemia, the amplified immune-inflammatory response via the PKM2-CREB1-CIITA pathway has been implicated^36^.Similar mechanisms may operate in the coronary intima and ischemic myocardial regions, exacerbating local inflammation and promoting plaque instability, thereby increasing the risk of MI. LOC124901307 belongs to the non-coding RNA (ncRNA) category; although its specific function remains unclear and studies are lacking, it may contribute to the occurrence and progression of MI by modulating gene expression or regulating pathways related to vascular homeostasis. Individuals with a history of tuberculosis exhibit a significantly increased risk of cardiovascular diseases, including MI, acute coronary syndrome, ischemic stroke, and peripheral arterial disease.^37^This may be related to the chronic inflammatory response, immune activation, and endothelial dysfunction induced by tuberculosis infection. Future studies could further elucidate its molecular mechanisms and clinical potential, providing novel strategies for the early diagnosis and targeted intervention of MI.

Although this study provides new insights into understanding MI, it still has certain limitations. First, it relies on publicly available GWAS data for MR analysis, which may be influenced by factors such as data quality, sample selection bias, and genetic heterogeneity. Secondly, the data used in this study are mainly derived from European populations, and the GWAS data for immune cells and metabolites are primarily from populations with relatively homogeneous genetic backgrounds and specific characteristics. Such homogeneity may limit the external validity of the findings, as differences in genetic architecture, gut microbiome composition, dietary habits, and lifestyle exist across populations. These differences may affect immune responses, metabolite levels, and susceptibility to MI, potentially leading to distinct causal relationships in non-European populations. In addition, due to the lack of information and reports on unknown metabolites, this study faces challenges in interpreting and discussing the MR analysis results. Future research should further collect individual-level data from MI patients to validate and expand the findings, while incorporating more diverse populations and considering the interaction of multiple factors to more comprehensively elucidate the pathogenesis of MI.

## Conclusion

In this study, mediation MR analysis was employed to systematically evaluate the causal relationships and mediation effects among 731 immune cell traits, 1,400 plasma metabolites, and MI. A total of 38 immune cell phenotypes and 96 plasma metabolites were identified to have causal associations with MI. The results demonstrated a significant positive causal relationship between HLA DR on Dendritic Cells and MI, while reverse MR analysis did not reveal any effect of MI on HLA DR on Dendritic Cells. Further mediation MR analysis indicated that HLA DR on Dendritic Cells may influence the development of MI through Deoxycholic acid glucuronide levels, Phosphate to N-acetylneuraminate ratio, and Glycosyl ceramide, with proportions of mediation effects of 7%, 4.6%, and 6.6%, respectively. Elevated levels of HLA-DR on dendritic cells may increase the risk of MI by decreasing deoxycholic acid glucuronide levels, increasing the phosphate to N-acetylneuraminate ratio, and elevating glycosyl ceramide levels. Through systematic analysis of SNPs associated with HLA-DR on dendritic cells and these three metabolites, we identified key genes, including NUBP1, TVP23A, RBFOX1, DNAAF5, CLVS1, FCGR3A, CIITA, LOC124901307, as well as the tuberculosis signaling pathway, which may mediate potential interactions between immune phenotypes and metabolic traits ( Central Illustration ). In summary, this study provides new insights into the pathogenesis of MI, offers additional evidence to elucidate its underlying mechanisms, and may serve as a reference for identifying potential therapeutic targets.
